# Male Genitoplasty for Intersex Disorders

**DOI:** 10.1155/2008/685897

**Published:** 2008-11-04

**Authors:** Shilpa Sharma, Devendra K. Gupta

**Affiliations:** Department of Pediatric Surgery, All India Institute of Medical Sciences, New Delhi 110029, India

## Abstract

*Aim*. To evaluate surgical procedures adopted for male genitoplasty in intersex disorders. *Patients and Methods*. Case records of intersex patients undergoing male genitoplasty from Pediatric Intersex clinic were studied. *Results*. Of 356 intersex cases undergoing urethroplasty from 1989–2007, the hypospadias was penoscrotal (68%), scrotal (17%) and perineal (15%). 351 patients underwent chordee correction for mild: moderate: severe chordee in 24 : 136 : 191 cases. Byars flaps were fixed upto the corona in 267 cases. Urethroplasty performed was Theirsch duplay in 335 cases, Snodgrass in 16 cases and Ducketts onlay graft in 5 cases that did not require chordee correction. Age at urethroplasty was 2.5 years—22 years (mean 11.5 years, median—5.6 years). Penoscrotal transposition correction and testicular prosthesis insertion were performed independently. Complications included fistula (45), recurrent fistula (11), stricture (12), baggy urethra (8) and recurrent infection due to persistent vaginal pouch (5). Additional distal urethroplasty was required in 15 patients for previous urethroplasty done upto the corona 5–15 years earlier. *Conclusion*. Hypospadias in intersex disorders is associated with severe chordee in most cases and requires an early chordee correction to allow phallic growth, staged urethroplasty and multiple surgeries to achieve good cosmetic and functional results.

## 1. INTRODUCTION

To perform urethroplasty for cases of intersex disorders, now designated as disorders of sex differentiation, it requires far sight into the problems that are faced even many years after the urethroplasty. A number of surgical procedures need to be performed before the final outcome is attained. These will be discussed in detail in this article along with the authors experience in this field.

The vital decision in cases of intersex disorders is the accurate diagnosis and the gender assignment. The gender assignment takes into account the prevalent social factors in a community and the parent's desire [1]. There are still some countries where the parents would prefer to have an inadequate male rather than an incomplete female.

With lot of global discussion on the patient's wishes when they attain puberty, it has now become important to keep a possibility of a change in gender in some cases and wait a little longer before a definitive surgery unless there is parental pressure. These controversial issues have long been debated and vary from community to community.

**Purpose**

This work aims to study the surgical procedures performed for male genitoplasty in cases of intersex disorders.

## 2. PATIENTS AND METHODS

Case records of 356 intersex patients undergoing male genitoplasty from 1989–2007 from the Pediatric Intersex clinic of the department of pediatric surgery, AIIMS, were studied. Preoperative investigations included chromosomal analysis, ascending urethrogram, abdominal ultrasonography, and hormonal work up to establish the diagnosis of intersexuality.

## 3. RESULTS

Of 526 patients that had undergone urethroplasty, 356 were cases of intersex disorders. These included 298 cases of male pseudohermaphrodite, 24 cases of True Hermaphrodite, 27 cases of mixed gonadal dysgenesis and dysgenetic male pseudohermaphrodite, and 7 cases of congenital adrenal hyperplasia.

**Figure 1:**
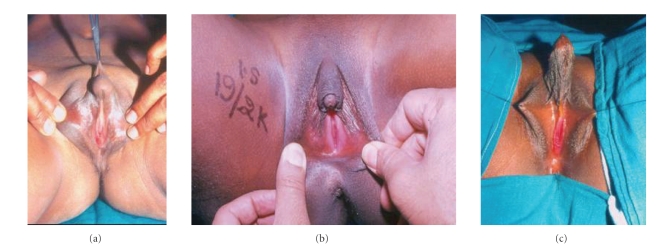
(a) A case of mixed gonadal dysgenesis with severe chordee, perineal hypospadias, and visible urethral and vaginal openings. (b) A case of intersex disorder with small phallus, fish mouth urethra, and mucosa-lined urethral plate. (c) Post chordee correction case of intersex disorder for second stage reconstruction with long urethroplasty.

**Figure 2:**
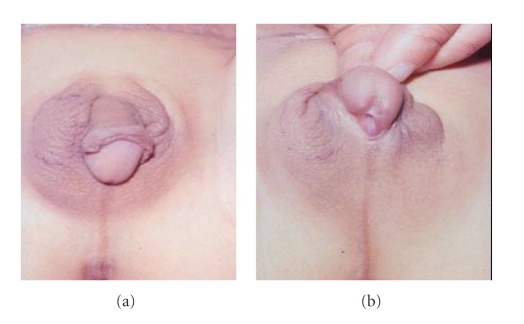
A case of True Hermaphrodite with (a) well-developed phallus and right scrotal ovotestis gonad, and (b) penoscrotal hypospadias and severe chordee.

The hypospadias was penoscrotal in 242 (68%) cases, scrotal in 61 (17%), and perineal in 53 (15%) cases (Figures 1, 2). The mullerian duct opening was present in the perineum along with the urethral meatus in 35 (10%) cases. A genitogram performed in all cases was sufficient to outline a vaginal pouch and establish the diagnosis of male pseudohermaphrodite in the presence of unilateral- or bilateral-descended gonads and absent uterus with XY karyotype.

Out of 356 cases, 351 patients had chordee and underwent chordee correction between 2 months—7.6 years age (mean 23.6 months). Five patients did not have chordee. The chordee was mild: moderate: severe in 24 : 136 : 191 cases. Byars flaps for the chordee correction were fixed upto the corona in 267 cases. Testosterone (5–10%) cream was advised for local application daily for 3 months at one time. The patients were called for follow up after that, and the response was assessed. If response was noted, it was continued for other three months. The treatment was stopped if any side effects were noted.

Periodic local treatment with testosterone was also advised later, during childhood till puberty, to help in penile growth in patients with low testosterone level and those having a small-sized phallus. However, systemic injections were given only postpuberty to prevent bone maturation if given earlier.

**Figure 3:**
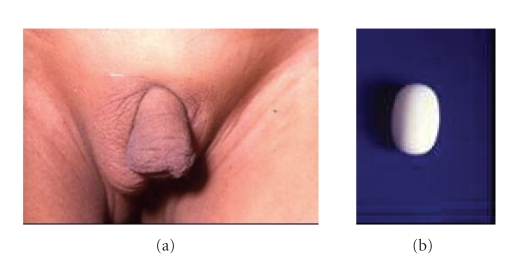
(a) A case of true hermaphrodite with right scrotal ovotestis and left undescended gonad needing a prosthesis. (b) Indeginized (DK Gupta) testicular prosthesis made of Teflon.

Theirsch Duplay urethroplasty was performed in 335 cases, Snodgrass urethroplasty was made in 16 cases, and Ducketts onlay graft was performed in 5 cases for whom chordee correction was not required. The age at urethroplasty was 2.5–22 years (mean 11.5 years, median—5.6 years). Scrotal transpositions for cases with penoscrotal transposition as well as testicular prosthesis were performed as an independent operation so as not to jeopardize the perineal and preputial flaps (Figure 3). The complications included fistula formation in 45 cases, recurrent fistula formation in 11 cases, stricture formation in 12 cases, baggy urethra in 8 cases, and recurrent infection due to persistent vaginal pouch in 5 cases. Postoperative infection was more in the boys that were operated after ten years of age. Additional surgery was performed for diverticuli in 3 cases. An additional distal urethroplasty was requested by 15 patients, where the urethral tube was constructed earlier only upto the corona 5–15 years following the previous urethroplasty. Five patients out of 356 (1.4%) developed hair growth in the region of the scrotal neourethral tube that led to infection and urinary obstruction. They had to undergo epilation or redourethroplasty with removal of the skin patch.

The urethroplasty has been completed in 324 patients and 27 patients are still awaiting the urethroplasty. Of the 46 cases that had attained puberty, 23 (50%) of them had to milk their urethra after voiding. Intramuscular testosterone was administered every month for cases having a low testosterone level and those having a small-sized phallus. None had residual chordee on interrogation of the patients. The cases in which the chordee correction had been delayed had a relatively small phallus than those in whom chordee correction had been done earlier. The postoperative urethrography revealed few urethral diverticulae and pouches in 5 cases that were otherwise asymptomatic. The uroflowmetry revealed low mean flow in 20 out of 46 cases. This was due to the fact that there is poor outflow resistance in these cases due to underdeveloped corpus spongiosum. Ten out of twenty cases interviewed were able to ejaculate, though the amount was less and 8 had to milk the ejaculate.

## 4. DISCUSSION

To manage disorders of sexual differentiation, a growing need is felt for extensive counseling, informed consent, and adherence to ethical and legal norms that protect the rights of the child as outlined in respective constitutions and a multidisciplinary approach [2].

The foremost step is the sex assignment. This should be based upon what is judged to be the most likely adult gender identity, diagnosis, genital appearance and surgical options, fertility, cultural pressures, as well as family dynamics and social circumstance, with preference given to psychosocial factors when the outcome is unpredictable [3]. However, the complex management of these patients must be individualized [3]. In this series, seven cases of CAH were reared as males as they had already been reared as males for a long time before they came for treatment

Gender assignment procedures have been questioned by intersex activists opposed to early genital surgery [4]. Western societies have a binary perspective on gender and this leads to a stigma on intersex cases [4]. Also, the current data challenges the past practice of sex reassignment, thus, a careful judgment is warranted [5]. The debate whether surgical genitoplasty affects gender identity in the intersex infant centres around which is more vital for development of gender identity, that is, the biological sex of a child or the sex in which a child is reared [6].

Hormonal and genetic factors may have a more important role in gender identity and sexual satisfaction than previously recognized, whereas the importance of phallus size to male gender identity and sexual satisfaction may have been overestimated [7].

The impact of androgen imprinting on the developing brain has also been debated. A neutral upbringing may induce psychosocial consequences that are more damaging than carefully considered neonatal sex attribution and concordant surgical genitoplasty [6].

The spectrum of iatrogenic harm to children with intersex characteristics has now become a legal issue [8]. Thus, a multidisciplinary approach involving pediatric surgeons, endocrinologist, and psychiatry is necessary, along with educational programmes that promote tolerance in society to variations in gender [4].

The most common disorder of sexual differentiation is male pseudohermaphroditism that comprises about 55–60% of all cases [9]. However, some series from endocrine centres have the largest number of cases as congenital adrenal hyperplasia [10]. The causes of male pseudohermaphroditism include 17 beta-hydroxysteroid dehydrogenase deficiency, 3 beta-hydroxysteroid dehydrogenase deficiency, 5 alpha-reductase deficiency, and idiopathic male pseudohermaphroditism [11].

The timing of performing the masculinisation procedures is still a controversy. However, most feel that the optimal time for external genital correction is 2 years of age [3, 9, 12]. However, in this series, most of the patients presented late after running from pillar to post for accurate diagnosis. Moreover, in recent years, traditional views regarding the management of infants with intersex conditions have been challenged [7].

Some authors perform urethroplasty only after obligatory testosterone treatment [10]. The application of testosterone helps to make the skin supple besides increasing the length and girth of the phallus. Though prior to 1980s, single-stage reconstruction was in vogue, with the passing time, it has been realised that single-stage reconstruction is associated with more complications in cases with intersex disorders [13]. These include fistula formation, complete stricture, and wound dehiscence. The reason is that the phallus in these cases is inadequate to support the formation of a 6Fr urethral tube formed from the inadequate preputial skin at a tender age.

In this series, the number of urethroplasties in intersex cases is higher than that in hypospadias patients as being a tertiary-level hospital, the cases are selected for operations. Only the difficult cases or those with more proximal hypospadias are dealt with after ruling out intersex disorders.

In this series, most of the cases were operated with a two-stage procedure. In the first stage, the chordee is corrected, followed by urethroplasty from the neopenile skin flaps after an interval of at least 6 months. The Byars flaps for the chordee correction are fixed upto the corona in most cases as the glans is very underdeveloped at the time of chordee correction. The parents are then advised to apply local testosterone. For adequate chordee correction, the urethral plate was divided during the first stage. The fibrous tissue was excised. Total mobilisation was done upto the base of the phallus. As a result, the meatus moved proximally 3–6 cms. The excision of all fibrous strands assures the complete removal of chordee. The intervening deficiency after adequate chordee correction was covered with preputial skin that was mobilised during degloving of the phallus. The authors used to perform Gittes' test to assess chordee in the intial cases but soon realised that it is not necessary in cases where there is no doubt of residual chordee in cases where the urethral plate has been cut.

The severe chordee that is present in most of the cases prevents adequate growth of the phallus. The deficiency of testosterone, either systemically or locally due to various enzyme defects present in these cases, prevents adequate growth of the phallus both in length and girth. The application of steroid cream after chordee correction facilitates proper growth of the phallus that forms a good base for the neourethra. Also, the penile skin in these cases is thin and lacks strength and good vascularity for proper healing.

Dihydrotestosterone (DHT) is beneficial for patients with 5 alphareductase deficiency that are unable to convert testosterone into DHT due to absence/default of receptors. Some of these patients respond with an increase in penile size when 25 mg/d of 2% DHT cream is applied to the external genitalia. However, anabolic effects may enhance hypoglycaemia and bone maturation. Adverse reactions include pruritus, erythema, allergic contact dermatitis, and burning.

Once the phallus size is adequate, urethroplasty is performed. Recurrence of chordee was assessed by artificial penile erection test of Gittes and McLaughlin at the time of stage 2 reconstruction. The most common procedure adopted is Theirsch Duplay urethroplasty. Some authors have performed a single-stage procedure using a transverse pedicled preputial island flap as an onlay, tubularization of the mucosa in the perineal area, and end-to-end anastomosis to a tube made from the pedicled prepuce [11].

However, a one-stage male genitoplasty for male pseudohermaphroditism is accompanied by a reasonable incidence of major complications [11]. Thus, the two-stage technique for male genitoplasty is preferable [12]. The two-stage approach for severe hypospadias without intersex disorders results in excellent function, cosmesis, and patient satisfaction after puberty, with no chordee in patients though minor voiding and ejaculatory problems are to be expected [14].

However, cases with intersex disorders have a deficient spongiosum and thus the urethral tube is poorly supported. About 50% of them have to milk their urethra after voiding to keep themselves dry.

In intersex patients, Mullerian duct remnants are not an unusual occurrence. The presence of a vaginal pouch (utriculus prostaticus) does not affect the urethroplasty. A genitogram performed in all cases is usually sufficient to outline a vaginal pouch and establish the diagnosis. In this series, the Mullerian duct remnants were removed, if present, in all patients assigned a male gender, only the vaginal pouch was preserved. The uterus, fallopian tubes, and upper part of the vagina were removed in all, and the lower part of the pouch was left as such in all cases. The vaginal pouch was not removed in any case of male pseudohermaphrodite.

There is no evidence that removal of utriculus and Mullerian remnants, which are asymptomatic, is necessary. Due to potential injury to continence mechanisms and for preservation of fertility (vas deferens often joins the utricle), it is better to reserve surgical treatment only to symptomatic cases.

However, there are others who prefer to remove a big or symptomatic utriculus prostaticus [15]. Due to the location of the pouch, a surgical removal from the perineal or sacral route is always risky. If at all required as in symptomatic cases, a laparoscopic removal is much safe. Authors prefer to include the opening of the utriculus in the urethroplasty. If the opening joining the urethra is narrow, the opening is widened endoscopically.

In a series of 47 boys, based on the symptoms and the size of the remnants, the structures were removed in 32 patients by extirpation done by perineal approach in younger asymptomatic children, by transperitoneal approach, by posterior sagittal pararectal approach, or by combined abdominal and perineal approach [15]. Complications like rectal or bladder injuries and temporary impotence after abdominoperineal extirpation may occur during attempted removal.

The presence of Mullerian duct remnants may occasionally lead to symptoms of urinary infection, urinary retention, or epididymitis. However, these are manageable with courses of antibiotics during acute episodes and preventable by executing a habit of milking out the contents after micturation. Thus, on comparing the risks with the benefits, it is wiser to leave the vaginal pouch as such. In this series, only 5 patients had problems due to the vaginal pouch. The rest remained asymptomatic. These were more frequent in the cases of congenital adrenal hyperplasia that preferred to be assigned a male gender.

There may be lack of ejaculation related to frequent intrautricular termination of the vas deferens [15]. Some patients with androgen insensitivity syndrome, that is, the other end of male pseudohermaphrodites who are initially assigned a female gender, may seek help for conversion into a male at a later date. For these cases, the neophallus may be created from sensate-free forearm flaps or regional abdominal flaps that exist [16]. The corporal tissue is preserved for sensations and placed at a suitable place in the perineum.

Most series are satisfied with their functional and cosmetic outcome of masculinising genitoplasty in patients with ambiguous genitalia raised as males [17]. Good results may be expected if the initial phallus size is adequate, especially those that have responded to local testosterone. The results are poor in cases with micropenis and minimal virilisation [13]. Intramuscular testosterone is administered every month to most cases with a low testosterone level. The uroflowmetry revealed low mean flow in 20 out of 46 cases in this series due to the fact that there is poor outflow resistance in these cases due to underdeveloped corpus spongiosum. Spontaneous puberty may be observed in true hermaphrodites raised as males [13]. Most cases have to undergo multiple genital surgeries to correct the appearance of their genitalia and/or to enable sexual functioning [18, 19].

To summarize, hypospadias in intersex disorders is associated with severe chordee in most of the cases and requires an early chordee correction to allow phallic growth. The issue of genital surgery in infancy remains controversial although many adult patients do concur that infancy is the best time for such procedures. Good anatomical and functional results are achieved better with the two-stage repair and intervening period of local testosterone application.

## References

[1] Gupta DK, Menon PSN (1997). Ambiguous genitalia—an Indian perspective. *Indian Journal of Pediatrics*.

[2] Maharaj NR, Dhai A, Wiersma R, Moodley J (2005). Intersex conditions in children and adolescents: surgical, ethical, and legal considerations. *Journal of Pediatric and Adolescent Gynecology*.

[3] Nabhan ZM, Lee PA (2007). Disorders of sex development. *Current Opinion in Obstetrics and Gynecology*.

[4] Duncan ND, Gabay L, Williams E, Dundas SE, Plummer N, Leake PA (2006). Hermaphroditism: cytogenetics, gonadal pathology and gender assignment: a case report. *West Indian Medical Journal*.

[5] Frimberger D, Gearhart JP (2005). Ambiguous genitalia and intersex. *Urologia Internationalis*.

[6] Nihoul-Fékété C (2005). Does surgical genitoplasty affect gender identity in the intersex infant?. *Hormone Research*.

[7] Nelson CP, Gearhart JP (2004). Current views on evaluation, management, and gender assignment of the intersex infant. *Nature Clinical Practice Urology*.

[8] Gurney K (2007). Sex and the surgeon's knife: the family court's dilemma... informed consent and the specter of iatrogenic harm to children with intersex characteristics. *American Journal of Law and Medicine*.

[9] Krstić Z, Perovic S, Radmanović S, Neclić S, Smoljanić Ž, Jevtić P (1995). Surgical treatment of intersex disorders. *Journal of Pediatric Surgery*.

[10] Coran AG, Polley TZ (1991). Surgical management of ambiguous genitalia in the infant and child. *Journal of Pediatric Surgery*.

[11] Chertin B, Koulikov D, Hadas-Halpern I, Farkas A (2005). Masculinizing genitoplasty in intersex patients. *The Journal of Urology*.

[12] Hecker WC (1982). Timing and technic of the surgical correction of childhood intersex genitals including the procedure for partial vaginal aplasia. *Zeitschrift für Kinderchirurgie*.

[13] Nihoul-Fékété C, Thibaud E, Lortat-Jacob S, Josso N (2006). Long-term surgical results and patient satisfaction with male pseudohermaphroditism or true hermaphroditism: a cohort of 63 patients. *The Journal of Urology*.

[14] Lam PN, Greenfield SP, Williot P (2005). 2-stage repair in infancy for severe hypospadias with chordee: long-term results after puberty. *The Journal of Urology*.

[15] Krstić Z, Smoljanić Ž, Mićović Ž, Vukadinović V, Sretenović A, Varinac D (2001). Surgical treatment of the Müllerian duct remnants. *Journal of Pediatric Surgery*.

[16] Sohn M, Bosinski HAG (2007). Gender identity disorders: diagnostic and surgical aspects. *The Journal of Sexual Medicine*.

[17] Farkas A, Koulikov D, Chertin B (2004). Masculinizing genitoplasty in male pseudohermaphroditism. *Pediatric Endocrinology Reviews*.

[18] Brinkmann L, Schuetzmann K, Richter-Appelt H (2007). Gender assignment and medical history of individuals with different forms of intersexuality: evaluation of medical records and the patients' perspective. *The Journal of Sexual Medicine*.

[19] Sharma S, Gupta DK (2008). Gender assignment and hormonal treatment for disorders of sexual differentiation. *Pediatric Surgery International*.

